# Maternal satisfaction with delivery service and associated factors among women who gave birth at public hospitals in Guji Zone, Southern Ethiopia

**DOI:** 10.1186/s12905-024-03069-0

**Published:** 2024-04-08

**Authors:** Endale Megersa Alemu, Abdene Weya Kaso, Girma Worku Obsie, Hiwot Zelalem Fessaha, Gebi Agero

**Affiliations:** 1 Department of Family Health Services, Guji Zone Health Office, Negelle Borena, Ethiopia; 2 Department of Public Health, College of Health Science, Arsi University, Asella, Ethiopia

**Keywords:** Client satisfaction, Childbirth, Labor, Healthcare, Mothers

## Abstract

**Background:**

Maternal satisfaction during delivery services is considered an important indicator of the quality of healthcare in a hospital setting and determines the uptake of services during subsequent pregnancies. However, there is limited information on the magnitude of women’s satisfaction during delivery services in the study area. Thus, this study aimed to assess factors associated with maternal satisfaction with delivery services among women who gave birth at public hospitals in Guji Zone, Southern Ethiopia.

**Method:**

A facility-based cross-sectional study was conducted at public hospitals in Guji Zone from December 1, 2020, to January 30, 2021. Two hundred forty-nine women who gave birth at public hospitals were recruited by a systematic random sampling technique. The collected data were entered into the Epi Info 7 software and then exported to SPSS Version 26 for analysis. A logistic regression model was employed to identify the association between independent variables and maternal satisfaction during delivery services. A P-value less than 0.05 and an Adjusted Odds Ratio with 95% CI was computed to determine the strength of the association between these variables.

**Result:**

In this study, 138(55.4%), 95% CI (49.1–61.7) women were satisfied with delivery. Mothers who delivered through cesarean section (AOR = 2.92, 95% CI: 1.34–6.33), privacy assured (AOR = 3.14, 95% CI: 1.76–5.59), shorter duration of labor (AOR = 2.82, 95% CI: 1.64–4.62), waiting time ≤ 30 min (AOR = 5.15,95% CI:1.99–13.32) and normal fetal outcome (AOR = 2.63, 95% CI:1.42–4.85) were associated with mothers satisfaction with delivery care services.

**Conclusion:**

The overall magnitude of women’s satisfaction with delivery services is low, which is below the national client satisfaction target of ≥ 85%. Factors such as mode of delivery, assured privacy, short duration of labor, waiting time ≤ 30 min, and good fetal outcome were significantly associated with women’s satisfaction with delivery services. Therefore, healthcare providers should provide better management during intrapartum childbirth or emergency obstetric care to improve fetal outcomes during delivery services. In addition, health facility managers should avail infrastructure that helps to maintain the privacy of women who give birth in the facility.

## Background

At the global level, approximately 287, 000 women died from maternal causes in 2020, with more than two-thirds of the deaths occurring in developing countries, especially sub-Saharan African (SSA) countries [1–3]. In Ethiopia, the maternal mortality rate (MMR) is 416 per 100,000 live births, accounting for 3–5% of all maternal fatalities worldwide [4]. Delays in deciding to seek care, getting care, receiving proper healthcare, having insufficient supplies and equipment, and poor quality of services are associated with higher rates of maternal death [5–7]. To reduce MMR and neonatal mortality, the availability of institutional delivery alone is insufficient, and understanding maternal perceptions of care and satisfaction with delivery services is essential [8, 9]. As a result, monitoring mother satisfaction during delivery services has gained more attention and has become a priority for health programmers and managers in the health sector [9–11]. The World Health Organization (WHO) also recommended respectful, women-centered, and evidence-based maternity practices to improve delivery service outcomes. It also suggests routinely evaluating women’s satisfaction with the care they get [12, 13]. In Ethiopia, the government has implemented a free maternal healthcare policy to reduce pregnancy-related mortality. However, maternal satisfaction with delivery services is still a serious concern [14–16]. The magnitude of maternal satisfaction with delivery services varies from facility to facility. For example, 81.7% of mothers in Debre Markos [17], 79.1% in Gamo Gofa [18], 87.7% in Hawassa City [19], 61.9% in the Amhara Region [20], and 65.2% in the Jimma zone [21] were satisfied during delivery services. Factors such as sociodemographic factors [22–24], respectful care services, cleanliness of health facilities [25–27], privacy measures [20, 28, 29], and the availability of laboratory services and drugs [17, 25, 30, 31] were reported to influence maternal satisfaction during delivery. Despite governments’ considerable attempts to promote institutional service delivery, health service quality is still inadequate and remains a significant problem in Ethiopia [32]. Maternal satisfaction assessment is thus an important indicator of the quality of healthcare in a hospital setting, as it determines the uptake of services during subsequent pregnancies [33, 34] Thus, determining mother satisfaction with maternity services plays a vital role when designing programs and quality improvement projects that improve maternal and child health services. It can also provide systematic information for service providers, local planners, and other stakeholders to understand how well the delivery service is performing according to client perceptions and what changes may be needed to meet client preferences. Therefore, this study aimed to assess factors associated with maternal satisfaction with delivery services among women who gave birth at public hospitals in Guji Zone, Southern Ethiopia.

## Methods and materials

### Study setting, design, and period

A facility-based cross-sectional study was conducted among 249 mothers who gave birth at a public hospitals in Guji zone between December 1, 2020, and January 30, 2021. The Guji zone is located in southern Ethiopia, 610 km away from Addis Ababa, the capital of Ethiopia. The zone has an estimated total population of 1,588,430 people, 443, 592 of whom were women of reproductive age. There are four public hospitals and 42 health centers that provide healthcare services in the Zone. This study was conducted at Negelle Borena General Hospital and Adola Woyu General Hospital. Negelle Borena General Hospital is located in southern Ethiopia, 610 km from Addis Ababa. The hospital has one hundred twenty-six (126) beds to deliver its healthcare services. According to a human resource department report (2020), the hospital is running outpatients, inpatients, and emergency services with ten doctors, one gynecologist, one surgeon, two Integrated Emergency Surgeons (IESO), ninety-six nurses, and other healthcare workers. The hospital has a catchment population estimated to be 321,346, of which 59,897 were women of reproductive age. In 2020, 4168 women were delivered at hospital based on the hospital service delivery report. Adola Woyu General Hospital is another hospital located 445 km from Addis Ababa, the capital of Ethiopia. Currently, the hospital has ninety-seven beds for the provision of basic health services. The hospital employed 98 nurses, one surgeon, three IESOs, ten midwives, thirteen doctors, one gynecologist, and other healthcare workers to provide outpatient, inpatient, and emergency care for the catchment communities. The catchment population of Adola Woyu Hospital is estimated to be 630,938, 117, 544 of which were women of reproductive age. According to the hospital service delivery report, 4612 women were delivered in the hospital in 2020.

### Study population

The source populations were all women who visited public hospitals in the Guji zone for delivery services whereas all randomly selected women who gave birth at selected public hospitals in the Guji zone were the study population.

#### Inclusion criteria

All women who gave birth at the selected public hospital within the study period were included.

#### Exclusion criteria

Those women who were unable to respond (e.g., who were unable to talk) or were very sick were excluded.

### Sample size and sampling procedure

A single population proportion formula was used to determine the sample size using the following assumptions, 95% confidence interval, 5% margin of error, 10% non-response rate, and 81.7% prevalence of maternal satisfaction [17]. The final sample included 253 mothers who delivered in public hospitals. Two public hospitals that provide basic and comprehensive obstetric and neonatal care were purposively included in the study due to reports of client complaints with the service delivery, especially around the delivery unit. Study participants were proportionally allocated to each hospital based on a review of the average two-month delivery service report for the year 2019/2020. The calculated sample size was proportionately allocated for each health facility, and the selection was performed using a systematic sampling technique. The number of clients in each health facility was determined by reviewing the two-month document of each health facility and the number of subjects to be included in the study from each health facility. The first participant was randomly selected using a lottery method on the first date, and every two women who delivered were recruited until the sample size was completed.

### Data collection tool and procedure

A standardized questionnaire, administered by an interviewer, was used to collect the data. The questionnaire collected data on women’s sociodemographic characteristics, obstetric-related factors, and structural and process-related aspects to determine maternal satisfaction. The mothers’ satisfaction levels were measured using questions that were adapted from the Standards for Hospitals’ Quality Assessment Tool (i.e., Labour and Delivery Satisfaction Index Questionnaire) [35, 36] and the Donabedian Quality Assessment Framework [37, 38], which uses a five-point Likert scale ranging from “very dissatisfied” to “very satisfied”. The data were collected in the postpartum unit. Two BSc nurses were recruited as data collectors under the supervision of two public health professionals.

### Data quality assurance

To ensure the consistency of the questionnaire, we translated the English version of the questionnaire to Afan Oromo and again back to English by a language expert. The questionnaire was pretested on 5% of the study participants at Negelle Health Center before the data collection and amendments were performed accordingly. Two days of training on objectives and ethical issues were provided for the data collectors and supervisors by the principal investigator before the study. The collected data were checked for completeness and consistency daily by the principal investigator before data entry.

### Variables of the study

Maternal satisfaction with delivery care service was the dependent variable. Variables such as sociodemographic/economic factors (age, religion, marital status, education level, mother’s occupation, residence, and monthly income), obstetric factors (parity, ANC, pregnancy status, duration of labor, mode of delivery, fetal condition and maternal outcome), structure related factors (physical environment, presence of awaiting area, comfort of the waiting area, service charge, availability of laboratory services, drugs and cleanliness of the toilet) and process and care-related factors (sex of care provider, waiting time, privacy, perceived provider competency, pain management, newborn care, support from staff in breastfeeding, asking permission throughout each procedure, politeness of healthcare provider, number of healthcare providers and promptness of care) were the independent variables.

### Operational definition

#### Maternal satisfaction with service delivery

This was expressed as a state of satisfaction with healthcare service uptake in the dimension of quality of care service which involves structural and process activities. Mothers’ satisfaction with the process and health institution aspects of delivery services were determined using 20 items on a five-point Likert scale ranging from very dissatisfied to very satisfied adapted from the Donabedian Quality Assessment Framework. Internal consistency was checked using Cronbach’s alpha coefficient, which was found to be 0.72. Since each item had a 5-point Likert scale ranging between 1 and 5, the maternal satisfaction scores were calculated by summing the answers to all the items. The overall maternal satisfaction was subsequently categorized into satisfied and dissatisfied using a threshold determined by the demarcation threshold formula, which is (total highest − score lowest score)/2 + total lowest score. Respondents who scored less than the cut-off point were categorized as “dissatisfied”, whereas those who scored greater than or equal to the cut-off point were categorized as “satisfied” [39, 40].

#### Normal maternal outcomes

Included the absence of postpartum infection, postpartum hemorrhage, and 3rd or 4th degree tears, uterine rupture, sepsis, vesicovaginal fistula, bladder rupture, wound dehiscence, anemia, hysterectomy, maternal near-misses, labor abnormalities, adverse birth outcomes, and maternal death [41].

#### Normal fetal outcomes

Were defined as a fetus who was delivered alive or well or lacks the following conditions(low birth weight, birth trauma, perinatal asphyxia, meconium aspiration syndrome, APGAR score < 7, early neonatal death, or stillbirth [41, 42].

### Data processing and analysis

The collected data were coded, cleaned, edited, and entered into Epi Info 7 software and then exported to SPSS version 26 for analysis. Descriptive statistics such as frequencies and percentages were computed to present data using tables and graphs. A logistic regression model was employed to determine the association between an independent variable and maternal satisfaction during delivery services. Variables with *p* < 0.25 in the bivariate logistic regression analysis were retained in the multivariable logistic regression model and an adjusted odds ratio with a 95% confidence interval was used to measure the strength of the association. A *p* < 0.05 indicated statistical significance. Finally, the fitness of the model was checked by using the Hosmer and Lemeshow goodness-of-fit test.

## Results

### Sociodemographic characteristics

A total of 249 women were interviewed, yielding a response rate of 98.4%. Of the study participants, 195(78.3%) were aged 21–34 years of age, with a median age of 25.0 years ranging from 17 to 37 years. Two hundred forty-six (98.8%) of women were married, whereas 151 (60.6%) of women attended primary education and above. The average monthly income of the respondents was 1864.91 Ethiopian Birr (Table 1).

**Table 1:** Sociodemographic characteristics of women who gave birth at public general hospitals, South Ethiopia, 2020/2021

Variables(*n* = 249)	Frequency	Percentage (%)
**Marital status**		
Married	246	98.8
Other*	3	1.2
**Ethnicity**		
Oromo	159	63.9
Amhara	52	20.9
Other**	38	15.3
**Occupation**		
Housewife	113	45.4
Farmer	57	22.9
Other***	54	21.7
Gov’t employee	25	10
**Religion**		
Protestant	139	55.8
Muslim	62	24.9
Orthodox	43	17.3
Other****	5	2.0
**Residence**		
Rural	128	51.4
Urban	121	48.6
**Income**		
≤ 1500	133	53.4
> 1500	116	46.6
**Level of education**		
No formal education	98	39.4
Primary education	56	22.5
Secondary education	67	26.9
College and above	28	11.2
**Maternal age**		
< 20	45	18.1
21–34	195	78.3
35–49	9	3.6

*Note* * : Single, Divorced, Widowed

**:Tigre, Somali, Gurage

***:Student, Private employee, Merchant, Daily laborer

****:Wakefata, Catholic

### Obstetric history of the respondents

Of 249 women interviewed, 120(48.2%) were primiparous, 227(91.2%) had ANC follow-up and 25(10.0%) of them reported an unwanted pregnancy. Concerning the reason to visit, 218(87.6%) of women had planned childbirth. Labor persisted for ≤ 12 h in 149(59.8%)of women and 216(86.7%) of women waited ≤ 30 min to be seen by healthcare providers. Out of 249 participants, 220(88.4%) women did not have delivery-related complications, and 216(86.7%)of them delivered live babies (Table 2).

**Table 2:** Obstetric characteristics of women who gave birth in public hospitals in Guji zone, South Ethiopia, 2020/2021

Variables(n=249)	Frequency (%)
Current pregnancy status	
Wanted	224(90.0)
Unwanted	25(10.0)
**Antenatal care follows-up**	
Yes	227(91.2)
No	22(8.8)
**Number of ANC follow-up**	
< 4	97(42.7)
≥ 4	130(57.3)
**Reason For Visit**	
Planned childbirth	218(87.6)
Referred for childbirth	31(12.4)
**Parity**	
Primipara	120(48.2)
Multi-para	82(32.9)
Grand multi para	47(18.9)
**Waiting time(in minutes)**	
≤ 30	216(86.7)
> 30	33(13.3)
**Duration of labor (In hours)**	
≤ 12	149(59.8)
> 12	100(40.2)
**Mode of delivery**	
Spontaneous Vaginal Delivery (SVD)	140(56.2)
Instrumental delivery	48(19.3)
Cesarean section	61(24.5)
**Maternal outcome**	
Normal	220(88.4)
With complication	29(11.6)
**Fetal condition**	
Normal	216(86.7)
With complication	33(13.3)

### Maternal satisfaction with delivery services

**Fig. 1:**
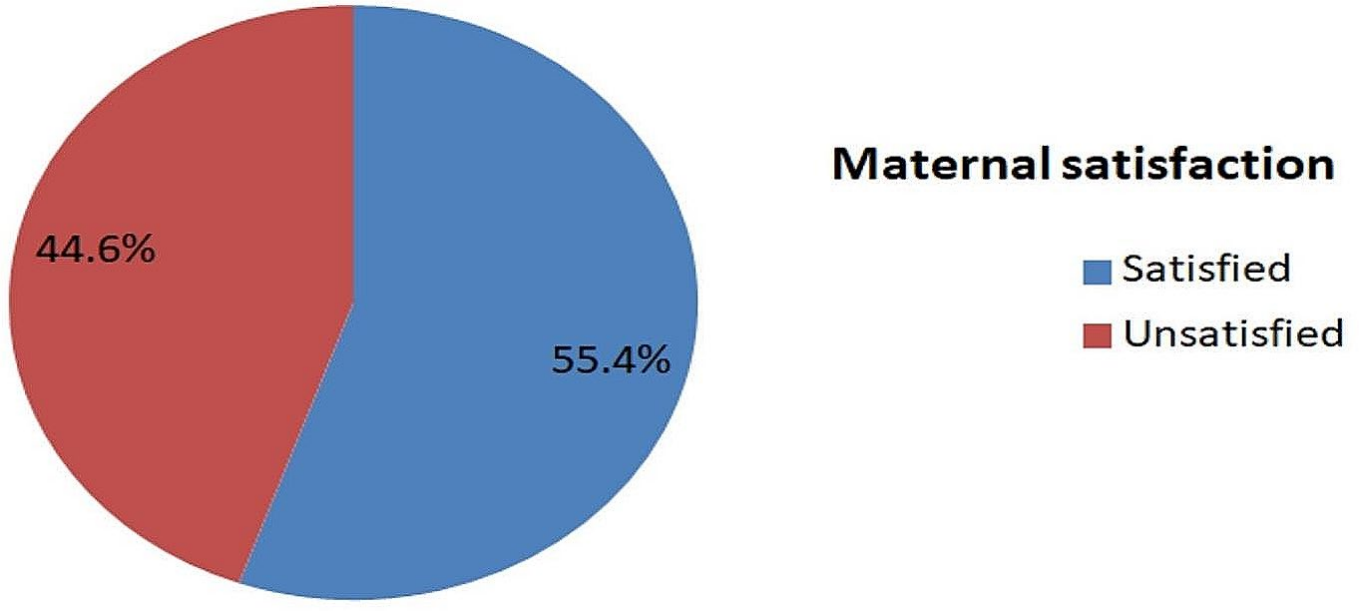
Overall maternal satisfaction with delivery services at a public hospital in the Guji zone

In this study, 138(55.4%), 95% CI (49.1–61.7) women were satisfied with delivery services (Fig. 1). In addition, the structure-related satisfaction of women during delivery services was 74.3% (95% CI: 68.6–79.5). A majority, 194(77.9%) of women were satisfied with the availability of adequate medical supplies and medications whereas 196 (78.7%) and 170(68.3%) of women were satisfied with the availability of the laboratory investigations and delivery room space, bed, and cleanliness respectively. The process-related maternal satisfaction during delivery services was 56.6% (95% CI: 50.4–62.7). One hundred eighty-six ( 84.7%) of women were satisfied with waiting time while 176 (70.7%) of the respondents were satisfied with how pain was managed during labor and delivery (Table 3).

**Table 3:** The responses of overall maternal satisfaction at public general hospitals, South, Ethiopia, 2020/2021

Satisfaction items	Very Dissatisfied	Dissatisfied	Neutral	Satisfied	Very Satisfied
Structural related Factor					
Number of Health Workers	27(10.8%)	19(7,6%)	10(4.0%)	139(55.8%)	54(21.7%)
Medical supplies and drugs	14(5.6%)	30(12%)	11(4.4%)	146(58.6%)	48(19.3%)
Sufficiency of Delivery room and bed	33(13.3%)	27(10.8%)	19(7.6%)	133(53.4%)	37(14.9%)
Laboratory Service	13(5.2%)	27(10.8%)	13(5.2%)	141(56.6%)	55(22.1%)
Availability and Sanitation of Toilet	15(6%)	34(13.7%)	14(5.6%)	137(55.0%)	49(19.7%)
Waiting area	19(7.6%)	48(19.3%)	12(4.8%)	134(53.8%)	36(14.5%)
**Process related factor**					
Waiting time	21(8.4%)	32(12.9%)	10(4.0%)	131(52.6%)	55(22.1%)
Helpfulness and politeness	18(7.2%)	66(26.5%)	5(2.0%)	126(50.6%)	34(13.7%)
Competency and confidence	24(9.6%)	24(9.6%)	5(2.0%)	162(65.1%)	34(13.7%)
Privacy measures	73(29.3%)	49(19.7%)	14(5.6%)	89(35.7%)	24(9.6%)
Sex of health workers	18(7.2%)	52(20.9%)	6(2.4%)	137(55.0%)	36(14.5%)
Permission before procedures	8(3.2%)	36(14.5%)	11(4.4%)	146(58.6%)	48(19.3%)
Explanation of the labor progress	11(4.4%)	36(14.5%)	7(2.8%)	155(62.2%)	40(16.1%)
Care whenever needed	11(4.4%)	24(9.6%)	4(1.6%)	154(61.8%)	56(22.5%)
Overall care	9(3.6%)	29(11.6%)	13(5.2%)	155(62.2%)	43(17.3%)
Question about your baby	20(8%)	40(16.1%)	7(2.8%)	152(61%)	30(12.0%)
Breastfeeding	12(4.8%)	27(10.8%)	4(1.6%)	147(59.0%)	59(23.7%)
Care and support for baby	11(4.4%)	29(11.6%)	8(3.2%)	145(58.2%)	56(22.5%)
Pain management	23(9.2%)	37(14.9%)	13(5.2%)	139(55.8%)	37(14.9%)
Encouragement	11(4.4%)	21(8.4%)	6(2.4%)	157(63.1%)	54(21.7%)

### Factors affecting maternal satisfaction with delivery services in hospitals

In bivariate logistic regression analysis, variables with a p-value less than 0.25 were considered to be candidates for multivariate logistic regression analysis. Accordingly, variables such as maternal educational level, mode of delivery, fetal condition, reason for visit, waiting time, pregnancy status, privacy assurance during delivery, sex of healthcare providers, and duration of labor were candidates for the multivariate logistic regression model. After controlling for confounding, the duration of labor, waiting time, fetal outcome, privacy status at delivery, and mode of delivery were found to be associated with maternal satisfaction during delivery. Women whose labor persisted for ≤ 12 h were almost three times more likely to be satisfied with delivery services than women whose labor persisted for more than 12 h(AOR = 2.82, 95% CI = 1.64–4.62). The odds of satisfaction with delivery among women who delivered through cesarean section were 2.92 times higher than those who delivered through spontaneous vaginal delivery (AOR = 2.92, 95% CI = 1.34–6.33). Women whose privacy was assured were almost three times more likely to be satisfied than women whose privacy was not assured (AOR = 3.14, 95% CI: 1.76–5.59). The odds of satisfaction with delivery services among mothers whose fetal conditions were normal were almost three times higher than those who delivered a foetus with complications (AOR = 2.63, 95% CI = 1.42–4.85). Women who stayed ≤ 30 min to be seen by healthcare providers were almost five times more likely to be satisfied than those who waited more than 30 min (AOR = 5.15, 95% CI = 1.99–13.32) (Table 4).

**Table 4:** Factor associated with maternal satisfaction with delivery service in public hospitals of Guji zone, South, Ethiopia, 2020/2021

	Maternal satisfaction status			
Variables	Satisfied (%)	Unsatisfied (%)	COR (95%CI)	AOR (95%CI)
**Maternal educational level**				
No formal education	50(51.0)	48(49.0)	1	1
Primary education	30(53.6)	26(46.4)	1.11(0.58-2.14)	0.93(0.422 − 2.03)
Secondary Education	37(55.2)	30(44.8)	1.19(0.64–2.21)	1.04(0.49–2.25)
College and above	21(75.0)	7(25.0)	2.88(1.12–7.39)	2.36(0.75–7.44)
**Modes of delivery**				
SVD	68(53.5)	72(47.6)	1	1
Cesarean Section	44(72.1)	17(17.9)	2.45(1.28–4.69)	2.92(1.34–6.33)*
Instrumental Delivery	22(42.6)	26(57.4)	0.80(0.42-1.54)	0.75(0.34-1.66)
**Fetal condition**				
Normal	127(58.8)	89(41.2)	2.86(1.32, 6.18)	2.63(1.42,4.85)*
With complications	11(33.3)	22(66.7)	1	1
**Reason for Visit**				
Planned	126(57.8)	92(42.2)	2.17(1.02–4.69)	2.24(0.89-5.61)
Referred	12(38.7)	19(61.3)	1	1
**Waiting time( in minutes)**				
≤ 30	128(59.3)	88(40.7)	3.35(1.52,7.38)	5.15(1.99,13.32)*
> 30	10(30.3)	23(59.7)	1	1
**Pregnancy status**				
Wanted	186(83.0)	38(17.0)	2.30(1.21–7.10)	2.70(0.92,7.93)
Unwanted	17(68.0)	8(32.0)	1	
**Privacy Assured**				
Yes	82(71.9)	32(28.1)	3.62(2.12–6.16)	3.14(1.76–5.59)*
No	56(41.5)	79(58.5)	1	1
**Sex of Providers**				
Male	63(46.0)	74(54.0)	2.18(1.31–3.64)	1.63(0.88–3.04)
Female	75(67.0)	37(33.0)	1	1
**Duration of labor(in hours)**				
≤ 12	95(63.8)	54(36.2)	2.33(1.39, 3.92)	2.82(1.64, 4.62)*
> 12	43(43.0)	57(57.0)	1	1

*Note* *=*P* < 0.05

## Discussion

Maternal satisfaction during delivery services is an important indicator of the quality of healthcare in a hospital setting [33, 34]Thus, this study aimed to assess factors associated with maternal satisfaction with delivery services among women who gave birth at public hospitals in Guji Zone, Southern Ethiopia. In this study, 55.4% (95% CI: 49.1–61.7) of women were satisfied with delivery services. This finding is in line with studies conducted in Kenya (54.5%) [43], Bahir Dar City, (61.4%) [22], and the Oromia Region, Ethiopia (55.35%) [44]. However, this finding is lower than studies conducted in Ambo town (83.9%) [29], Gamo Gofa (79.1%) [18], Debre Markos (81.7%) [17], Adama town (74.8%) [45] and higher than studies done in West Shewa Zone (36.6%) [32], and Mizan Aman Town (30.4%) [46]. The possible reason for the discrepancy might be related to differences in the quality of services provided, study period, methods of measurement, cut-off point and tool used, and type of setting.

In this study, Women who delivered through cesarean section were more likely to be satisfied with delivery service than women who delivered through SVD. This finding is supported by studies conducted in Guatemala, Mexico, and Panama [47], Debre Markos [17], Nekemte, Ethiopia [48], and the Gamo Gofa Zone [18]. This could be because women who delivered through SVD experience prolonged labour which inflicts pain. However, those who are delivered by CS get relief from pain during labor and delivery by anesthesia used for the operation. In addition, shorter waiting time in women who gave birth by CS also results in a better fetal outcome, especially in a distressed foetus [49, 50]. The odds of satisfaction with delivery services among women whose privacy was assured were almost three times higher than their counterparts. This finding is in line with studies conducted in Mekelle, Ethiopia [51], and Nekemte, Ethiopia [48]. This might be due to the fact that women whose privacy was not assured during the physical examination of their reproductive organs feel ashamed and discomfort, which reduces their level of satisfaction with the services provided.

The odds of satisfaction with delivery services among women whose labour persisted for ≤ 12 h were almost three times higher compared with women whose labour persisted greater than > 12 h. This is consistent with studies from Debre Markos [17], Mizan Aman [46], Wolatia zone [52], and Nekemte, Ethiopia [48]. This is most probably due to prolonged labour which exposes women to severe pain and repeated vaginal examinations that cause discomfort to mothers. In addition, prolonged laboring time also exposes women to stress/fear about their birth outcome, which reduces their level of satisfaction with delivery service. The Likelihood of satisfaction with delivery service was almost five times higher among women who waited ≤ 30 min to be seen by the healthcare provider compared with women who waited for > 30 min. This is similar to a study conducted in Debre Markos [17], Nekemte Ethiopia [48], and Adama Town [45]. This might be because women with prolonged waiting times might perceive that healthcare providers ignored them to provide appropriate and timely services which leads to dissatisfaction with the service provided. Moreover, women who waited for a longer period to receive services offered had a high probability of facing poor fetal outcomes such as stillbirth, and newborn complications which can also lead to clients’ discontent [53]. Fetal outcomes have also shown a significant association with women’s satisfaction with delivery services. A participant whose fetal outcome was normal were almost three times more likely to be satisfied than their counterparts. This is consistent with studies conducted in Ambo town, Ethiopia [29], Nekemte, Ethiopia [48], and Amhara region, Ethiopia [20]. This is might be due to the fact that women want to deliver a healthy baby; therefore, if she faces stillbirth or delivers a baby with complications, the mother might perceive that it is due to the healthcare provider’s negligence or faults, which leads to dissatisfaction with delivery services.Even though our study has a good response rate and the results can be generalized to other similar settings, it is not free from limitations. First, we interviewed women in the postpartum ward to determine their level of satisfaction with delivery services. Therefore, result of this study can be overestimated due to social desirability bias as mother might be reluctant to disclose their true feelings about the services provided. Second, our study used only quantitative cross sectional study design and was not supported by qualitative data. This limited us to explore other factors that have potential to influence maternal satisfaction with delivery services. Despite we expected difference in quality of service provided at day or night, the study failed to account time variation in analysis to indicate at which time delivered women was more satisfied with services provided.

## Conclusion

Only 55.4% of women were satisfied with delivery services, which is below the national client satisfaction target of ≥ 85%. Factors such as mode of delivery, assured privacy, short duration of labor, waiting time ≤ 30 min, and good fetal outcome were significantly associated with women’s satisfaction with delivery services. Therefore, healthcare providers should provide better management during intrapartum childbirth or emergency obstetric care to improve fetal outcomes during delivery services. In addition, health facility managers should avail infrastructure that helps to maintain the privacy of women who give birth in the facility.

## Data Availability

The data set used during analysis for the study is available from the corresponding author upon reasonable request.

## Abbreviations

AOR: Adjusted odds Ration
ANC: Antenatal Care
CS: Cesarean Section
CI: Confidence Interval
FMOH: Federal Ministry of Health
COR: Crude odds Ratio
LMIC: Low and Medium-Income Countries
MMR: Maternal Mortality Ration
SD: Standard Deviation
STD: Sexually Transmitted Disease
STI: Sexually Transmitted Infection
SSA: Sub Saharan African
SVD: Spontaneous Vaginal Delivery
WHO: World Health Organization
